# Collective Dynamics of Active Cytoskeletal Networks

**DOI:** 10.1371/journal.pone.0023798

**Published:** 2011-08-26

**Authors:** Simone Köhler, Volker Schaller, Andreas R. Bausch

**Affiliations:** E27 Cellular Biophysics, Technische Universität München, Garching, Germany; Swiss Federal Institute of Technology Zurich, Switzerland

## Abstract

Self organization mechanisms are essential for the cytoskeleton to adapt to the requirements of living cells. They rely on the intricate interplay of cytoskeletal filaments, crosslinking proteins and molecular motors. Here we present an *in vitro* minimal model system consisting of actin filaments, fascin and myosin-II filaments exhibiting pulsatile collective dynamics and superdiffusive transport properties. Both phenomena rely on the complex competition of crosslinking molecules and motor filaments in the network. They are only observed if the relative strength of the binding of myosin-II filaments to the actin network allows exerting high enough forces to unbind actin/fascin crosslinks. This is shown by varying the binding strength of the acto-myosin bond and by combining the experiments with phenomenological simulations based on simple interaction rules.

## Introduction

The cytoskeleton of eukaryotic cells is a highly flexible and adaptable scaffold that undergoes constant remodeling to meet their changing needs. With its unique static and dynamic properties it facilitates tasks as complex and diverse as cell division, cell locomotion or phagocytosis. Intracellular patterns can emerge, as has been observed in the apical constriction during Drosophila gastrulation, where the tissue rearrangement is driven by pulsed contractions of the actin myosin cytoskeleton [1], [2]. Similar self organization mechanisms are of outmost importance in many aspects of cellular development [3]. All these processes rely on the intricate interplay between three major components: actin filaments, molecular motors and crosslinking proteins. While a polymer network consisting of filaments and crosslinkers result in a viscoelastic physical gel, molecular motors exert local forces and turn it into an active gel [4]. The dynamics in these active actomyosin gels can be coordinated in time and space as has been observed for the pulsed constrictions during dorsal closure giving rise to a collective behavior *in vivo* [5].

A processive force exertion of the molecular motors inside a purely viscous environment would lead to ballistic motion of the cytoskeletal material, only limited by the viscous friction of the environment. Inside of a homogenous viscoelastic medium additional relaxation processes occur, which would mainly result in a slowdown of the dynamics.

Yet, the cytoskeleton is far from being homogeneous. Here, the coordinated action of molecular motors and crosslinking proteins leads to the formation and stabilization of a wide variety of structures that in turn affect the large scale dynamics of the system. It is the feedback between structure formation and dynamic properties that sets the functional properties of cytoskeletal systems and active gels in general. However, due to the lack of adequate model systems, it remains difficult to address these feedback mechanisms.

Force dissipation in the viscoelastic environment and the inherent heterogeneity should modulate the dynamics in a nontrivial way: The transport by molecular motors relies on the interaction with discrete and anisotropic filaments, which could suggest the occurrence of anomalous diffusion processes. At the same time, the force exertion inside of crosslinked cytoskeletal networks are expected to induce a force rate dependent rupturing of the crosslinking points [6] and thus the locally induced changes of the elastic environment will also affect and modulate the transport and structure formation dynamics. Thereby, it would be conceivable that the connectivity of the filamentous network enables the coordination of the transport and reorganization processes resulting in the appearance of collective modes.

To shed light on the principles underlying the physics of such active gels, to examine their microscopic dynamics and to classify the thereby resulting dynamic structures, we study a reconstituted actin network that is actively set under stress by molecular motors [7]. While crosslinking proteins are required for the mechanical stability of the actin network, molecular motors introduce an active component to the networks [8], [9], [10], [11]. The presence of only motor filaments and ATP does not suffice to induce any reorganization or structure formation in an actin solution. Only the combination of motors and crosslinking molecules can result in structure formation [12] and even can induce a macroscopic contraction of cytoskeletal *in vitro* networks [13], [14], [15].

Here we show that in an active and crosslinked network pulsatile collective modes and superdiffusive transport phenomena evolve, that depend critically on the interaction strength of the myosin-II motor filaments. Our recently introduced minimal approach [7] using a reconstituted actin/fascin network in presence of myosin-II filaments enables a backtracking of the pulsatile collective dynamic properties to the structure formation: The connectivity of the network is the basis for the coordinated reorganization of the cytoskeletal structure in the steady state. The dynamics are dominated by the complex competition of crosslinking molecules and motor filaments in the network. The key player is the relative strength of the binding of myosin-II filaments to the actin network: only a high binding affinity allows exerting high enough forces to unbind actin/fascin crosslinks resulting in network activity. The resulting dynamics can be described by an anomalous diffusion of the network's constituents.

## Results

**Figure 1:**
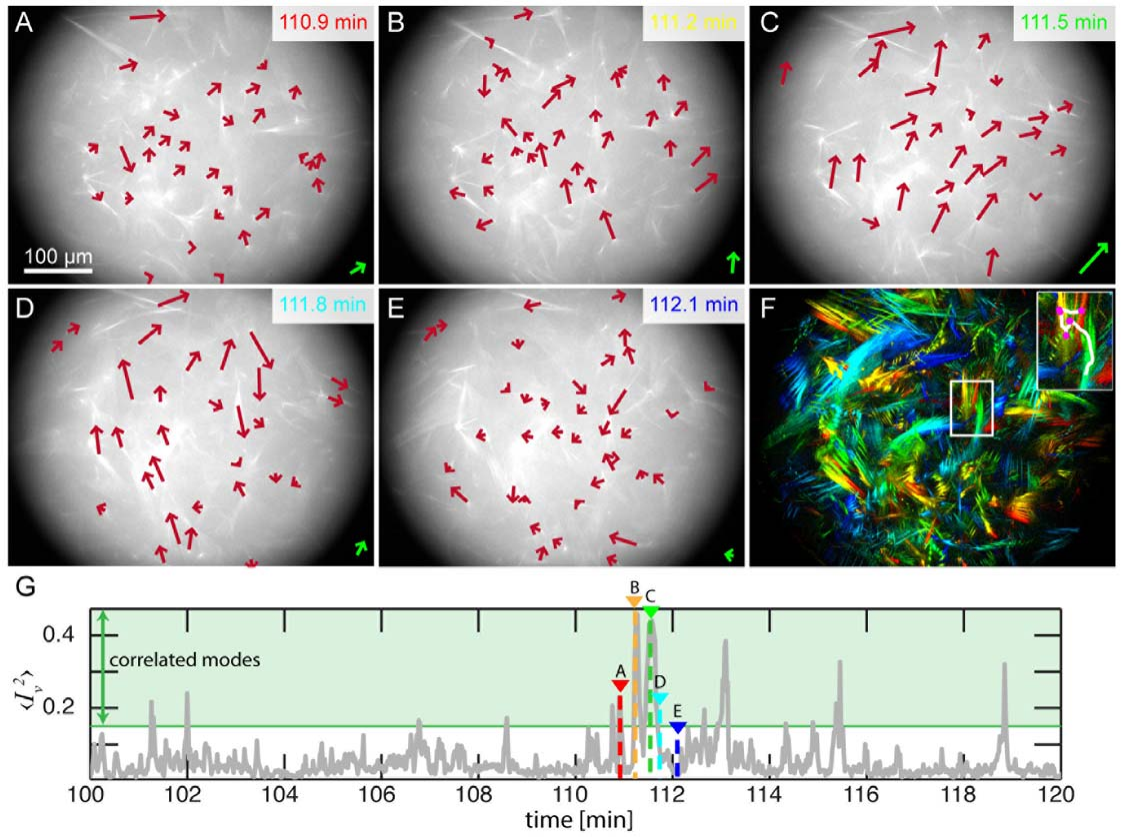
Structure and dynamics of actin/fascin/myosin networks. Fluorescence micrographs (**A–E**) of active actin networks at indicated times after initiation of polymerization (0.1 mM ATP) show the dynamic reorganization within the network. Red arrows indicate the movement of individual points in the network with lengths 20-fold magnified and a time-average over three frames. Green arrows show the resulting overall movement in the field of view (lengths are 40-fold magnified). These long range reorganization processes are summarized in the colored time overlay (red to blue, **F**). Due to the low magnification used in this experiment, very small structures cannot be resolved. Thus, the connectivity between the structures is higher than can be seen in these fluorescence micrographs. The trajectory of an individual structure exhibits persistent runs (inset, white line) intermitted by stall periods (inset, magenta dots). These run and stall phases are not only observed for individual structures, but also the whole network in the field of view exhibits pulsatile collective dynamics with movements being coordinated in time and direction: In stall phases, individual structures move predominantly for only short periods and in random directions (**A,E**). The stall phases are followed by periods with high activity in the entire network. During these run phases, the better part of the network shows long, persistent runs (**B,C**). The degree of coordination of the movement is measured by the average squared velocity cross-correlation function 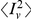, in which phases of collective movement are reflected as peaks. In such collective phases that last for up to 30 sec, the majority of the identified structures moves in approximately the same direction (**G**).

Active gels composed of 1 M actin filaments, 1 M fascin crosslinker and 0.1 M skeletal muscle myosin-II filaments in presence of ATP undergo drastic structural rearrangements. The dynamics within the network is characterized by a succession of persistent runs and stalling events of individual actin/fascin/myosin-II structures (fig. 1A–F and Movie S1). In general, the individual structures move independently. Yet, for short time intervals of about 30 s they can also syncronize their movements. These pulsatile collective modes are characterized by a coordinated movement in time and space of a few or even up to nearly all visible structures of the network (fig. 1G). Structures which are up to hundreds of microns apart are synchronized in their movement: during run phases their directional motion is coordinated (fig. 1C) and the active movement starts and stops almost simultaneously. This coordinated movement is not altering the steady state of the system and is not linked to a macroscopic contraction, which only occurs at much higher actin concentrations [7]. The long range coordination of the dynamic reorganization suggests the existence of a connectivity in the dynamic actin network. This connectivity is a prerequisite for the molecular motors to transport and exert forces between filaments. Thus, it is the connectivity and discrete nature of the network together with the force exertion mechanism, which enables the collective motion to occur. Introducing the velocity cross-correlation function 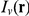 collective movements can directly be quantified. For each time point the average of the squared correlation function 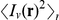 reflects the level of correlation in the system. High values of 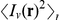 relate to a highly collective movement, while low values close to zero indicate intervals lacking collective modes (fig. 1G and fig. S1). At 0.1 mM ATP, for a correlation cutoff 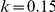 about 9% of the frames show correlated movement (see material and methods and movie S1).

**Figure 2:**
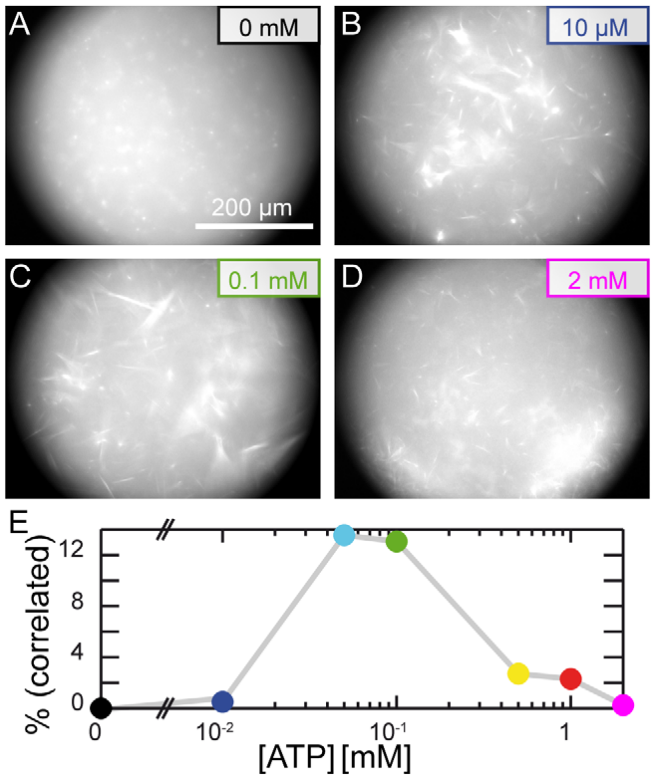
Dependence of the dynamics on the ATP concentration. In abscence of ATP, myosin-II filaments act as nucleation seeds, resulting in a multitude of small, well separated clusters as illustrated in the fluorescence micrograph 90 min after polymerization (**A**). In prescence of low (**B**), intermediate (**C**) or high (**D**) ATP concentrations, a highly dynamic network of larger structures is formed. The degree of correlated motion determined by the percentage of frames correlated more than 0.15 is dependent on the ATP concentration: Only intermediate ATP concentrations allow for highly correlated movement (**E**).

Microscopically, stall phases can be ascribed to the presence of the crosslinking molecules while the runs are the direct consequence of the activity of the myosin-II filaments. Accordingly, the dynamics and their collective modes should be set by the motor activity. Consequently, lowering the motor activity by decreasing the ATP concentration and thus lowering the motor velocity should lead to a continuous decrease of the network dynamics. Yet, the variation of the ATP concentration results in a more complex behavior, as the competition of the motors with the crosslinking molecules comes into play. We observe four distinct regimes (fig. 2): (i) in the absence of ATP a passive actin/fascin/myosin network consisting of small, diffusing clusters is observed (fig. 2A and Movie S2). Their movement is not correlated (fig. 2E). The passive actin/fascin/myosin network structure is distinct from an actin/fascin bundle network. This can be explained by a competitive binding of fascin and myosin-II to the actin filaments. Moreover, myosin-II filaments seem to act as nucleation seeds of the actin polymerization. (ii) At low ATP concentrations (10 M, fig. 2B) the low motor activity is limiting the number of runs and the stalls of the network structures are dominating the low dynamics. Consequently, collective modes barely occur in this crosslinker dominated regime. (iii) In the intermediate regime (up to 0.1 mM, fig. 2C) the highest correlation is observed. (iv) At high ATP concentrations (fig. 2D and Movie S3) correlated motion is again rarely observed. Moreover, at high ATP concentrations the networks become disrupted and huge clusters emerge. The maximal area of these clusters typically exceeds the area of a compact disc with radius of 80 m whereas structures at lower ATP concentrations remain significantly smaller.

The existence of an optimal ATP-concentration for the emergence of collective modes can be explained by the acto-myosin binding strength: Increasing the ATP concentration not only results in an increase in myosin-II sliding velocity but also decreases the maximal force myosin-II filaments can exert [16], [17]. The lower affinity of ATP-myosin to actin filaments limits the overall processivity of the myosin-II filaments. Thus the forces myosin-II filaments can exert are lower at high ATP concentrations than at intermediate concentrations of ATP. As a consequence, forced unbinding is less likely at higher ATP concentrations. Accordingly, large aggregates with many crosslinkers bound cannot be disrupted while small structures with only a few crosslinking points are still ruptured and rearranged. As a consequence, material is transported from small to large and highly crosslinked structures. This is reflected in the observed cluster formation. As the material is accumulated in these clusters the connectivity between them decreases resulting in the observed low correlation. At very low ATP concentrations the maximal force exertion of myosin-II decreases again [16] consistent with the observed decrease in dynamics.

**Figure 3:**
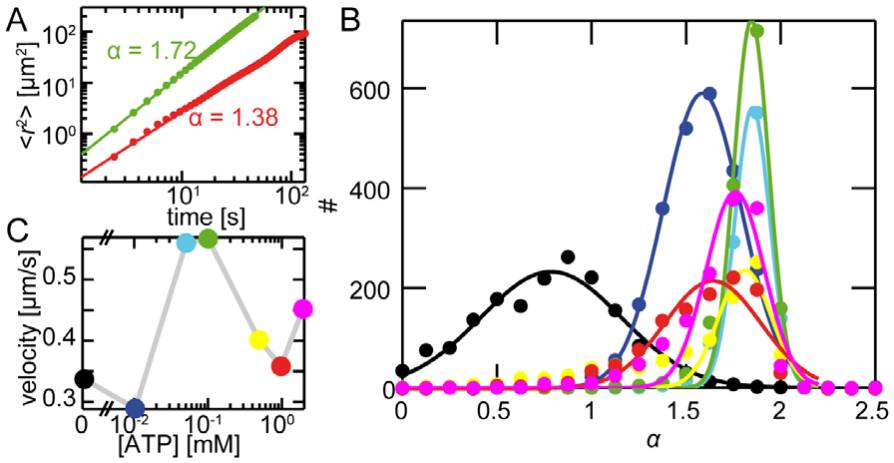
Superdiffusivity in active actin networks. Mean square displacements of two individual structures at 2 mM ATP show different but superdiffusive power law exponents (**A**). The distributions of the mean square displacement powerlaw exponents 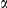 show maximal superdiffusivity at intermediate ATP concentrations (**B**, 50 (cyan) to 100 M (green); solid lines show gaussian fits). At low ATP concentrations (10 M, blue) the low activity of myosin-II limits the superdiffusive behavior. At high ATP concentrations (0.5 (yellow), 1 (red) or 2 mM (magenta)), myosin-II filaments are not able to exert high enough forces for constant network reorganization resulting in a lower mean superdiffusivity. Similarily, the highest mean run velocities are reached at intermediate ATP concentrations (**C**).

The dynamics of the individual structures in the network is best described by the mean square displacement [7], which is computed for about 1500 individual points per sample. For Brownian diffusion, the mean square displacement, 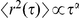, is expected to increase with time 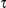 with a power law exponent 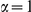 while 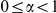 or 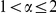 are indicative of sub- or superdiffusion, respectively [18], [19]. In presence of ATP, a clear superdiffusive behavior of individual clusters is found: the mean square displacements increase in time with 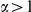 (fig. 3A). This can be traced back to the complex alternation of runs and stalls of the individual network structures. Due to similar durations of run and stall times, the m.s.d. is described by a single power law (fig. 3A). The distribution of the powerlaw exponents depends on the ATP concentration (fig. 3B): The passive state (i) is characterized by a subdiffusive behavior which can be attributed to the network hindering the diffusion. Already at low ATP concentrations (ii), the dynamics become superdiffusive. This superdiffusivity is even more pronounced at intermediate ATP concentrations (iii). However, at high ATP concentrations (iv) again a lower superdiffusivity is observed. Importantly, the width of the distribution increases at high ATP concentrations. This can be attributed to an increase of heterogeneity in the sample. These heterogeneities predominantly occur in this high ATP concentration regime. Similarly to the elimination of pulsatile collective modes, the lower superdiffusivity can be attributed to longer stalls (fig. S2) due to a decrease in forced unbinding events resulting in less activity and a larger variability.

The maximal velocity of individual structures in run phases of 0.6 m/s is observed for intermediate ATP concentrations while higher or lower ATP concentrations result in a decrease of the velocity in accordance with the results obtained for the pulsatile collective modes or superdiffusivity. In all regimes the velocities in run phases are an order of magnitude lower than the velocity of myosin-II filaments observed in gliding assays at similar ATP concentrations [20]. Thus, in all cases the speeds are not limited by the maximal speed of the motors, but by the presence of crosslinking bonds.

**Figure 4:**
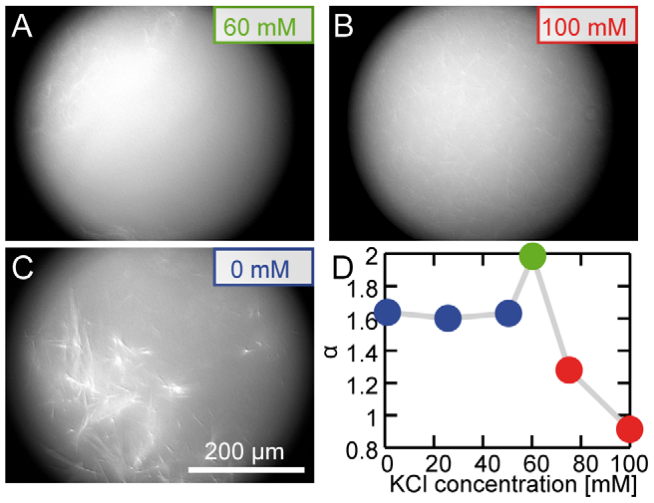
Dependence of the dynamics on the KCl concentration. Above a critical KCl concentration of 60 mM (**A**), an active actin/fascin/myosin network cannot be distinguished from a passive actin fascin network (100 mM, **B**), while below the critical concentration an active network is formed (**C**). The fluorescence micrographs are taken 90 min after initiation of polymerization. The superdiffusivity as characterized by the mean mean square displacement power exponent 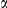 decreases above a critical KCl concentration of 60 mM (**D**).

Alternatively to the variation of ATP concentration, the crossbridge strength of the myosin-II filaments can be altered independently by the ionic strength [21]. An increase of the KCl concentration up to 200 mM does not affect the myosin-II filament length [22]. We also do not observe any differences in actin/fascin network structure upon variation of the ionic strength. Yet, exceeding a critical KCl concentration of 60 mM prevents any dynamic reorganization within the active gel (fig. 4): the structure of an active actin/fascin/myosin network at more than 60 mM KCl cannot be distinguished from a passive actin/fascin network. This critical concentration has also been observed in a gliding assay, where salt concentrations higher than 60 mM do not support motility of actin filaments [23]. At this ionic strength the affinity of ATP-myosin does not allow for permanent binding of the myosin-II to the actin filaments throughout its chemomechanical cycle. Similarily, decreasing the myosin concentration to 20 nM results in a quasi-static active gel [7] demonstrating the necessity of sufficiently high motor activity to induce a highly dynamic state in the active actin network.

Thus, the observed network reorganization, the emergence of collective modes and the superdiffusive behavior critically depend on the strength of the myosin-actin bond: Only a maximal binding strength of the myosin-II filaments to actin at low ionic strength allows exerting high enough forces to disrupt the actin/fascin crosslinking points and to reorganize the network structure. The network reorganization and the observed superdiffusivity not only rely on the competition between active and passive crosslinks but also on the respective binding strengths and affinities.

The good accessibility of all relevant system parameters within this reconstituted approach enables us to test the identified principles in a simple phenomenological simulation that is based on probabilistic interaction rules. Fascin bundles are modelled as polar rigid rods in a quasi two dimensional geometry they are monodisperse and are not subjected to rupturing events. The latter simplification is in accordance with experimental findings where single bundles are the smallest building blocks of cluster formation [7]. The bundles are propelled and crosslinked reflecting transport and passive binding processes. Both active and passive binding processes are subjected to forced unbinding events.

**Figure 5:**
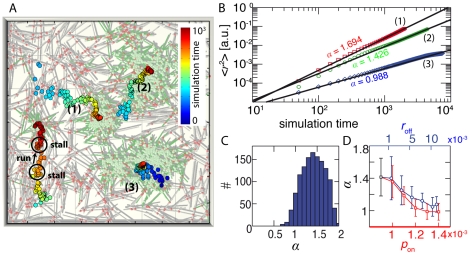
Simulation results. (**A**) shows a simulation snapshot superimposed with the time-resolved center-off mass positions of the respective clusters. As can be seen from the center-off-mass trajectories, individual clusters move in a succession of runs and stalls and in general move less the larger they are. This is reflected in the mean-square displacements depicted in (**B**): while the large cluster shows a diffusive motion (curve 3) the other clusters move in a superdiffusive manner with mean square displacement exponents 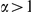. The heterogeneity in the system leads to a broad distribution of exponents 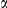 with a mean at 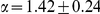 (**C**). The exact value of the mean exponent depends on the details of the motor- and crosslinker (un-)binding kinetics. Increasing the motor off-rate 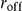 leads to a decreased activity in the system, visible in the decline of the mean square displacement exponent 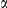 (**D**, blue curve). Likewise, the activity and hence the exponent 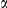 decline, if the crosslinker on-rate 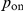 is increased (**D**, red curve).

The implementation of these basic processes already suffices to obtain a network dynamics strongly reminiscent to the experimental findings. Due to the concerted action of molecular motors, clusters are constantly transported and the trajectories they perform are characterized by a succession of runs and stalls (fig. 5A). During run phases the velocity of the fastest clusters does not exceed one tenth of the velocity of the motor proteins; and the larger the clusters are, the lower is their susceptibility to motor induced displacements and the less they move (fig. 5B). During stall phases clusters essentially exhibit a diffusive motion. In accordance with the experiment, runs and stalls average to a superdiffusive behavior, as can be seen in the distribution of exponents of the mean square displacement in fig. 5C. While this is in accordance with time dependent ensemble averages [7], the occurence of broad distributions directly relates to the intrinsic degree of heterogeneity in the system. Like in the experiment, the dynamics of individual clusters relies on local influencing factors like the local network connectivity, the local concentration of active crosslinks and the individual cluster size.

The exact values of 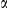 depend on the details of the actin-fascin and actin-myosin interaction. In the simulation, this is governed by the motor and crosslinker on- and off-rates. An increase in the motor off-rate 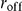 lowers the crossbridge strength and corresponds to the addition of KCl in the experiment. Similar to the experiment the increase of the motor off-rate hinders the reorganization in the system and individual structures, single rods or entire clusters move less. This is reflected in a reduced exponent of the mean square displacement, which successively decreases with increasing motor off-rate (fig. 5D). If the motor activity drops below a critical value, the dynamics becomes subdiffusive. In this regime the motor proteins are not strong enough to induce any significant network reorganization and the system essentially consists of one single cluster that only shows diffusive motion. A similar behavior is found if the on-rate of the crosslinking proteins 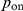 is increased. The higher the on-rate, the more the cluster dynamics is shifted to stall phases and the exponent 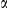 decreases concomitantly (fig. 5D).

Importantly, the simulation cannot retrieve the pulsatile collective run and stall phases observed in the experiment, where remote structures move in a coordinated manner (fig. 1). This indicates that the experimentally observed dynamics not only depend on the identified microscopic interactions but are modulated by a weak but far reaching connectivity within the system. While the incorporation of active transport, crosslinking and forced unbinding suffices to explain the superdiffusive transport properties, the simulation cannot account for long range force percolation as the force balance is only local with a typical range of a rodlength. This consistent with the experimental findings, as a lack of connectivity observed for high ATP concentrations leads to a decline in the proportion of collective modes (fig. 2E). Variuos generalizations of the model system are conceivable. One possibility would be to add a background network, modelling the weak but far reaching connectivity in the system that governs the emergence of collective modes. Another limitation of the simulation, is the monodispersity of the bundles in the simulation.

## Discussion

A minimal model system consisting of actin filaments, crosslinking molecules and myosin-II motor filaments exhibits drastic structural rearrangements that in turn affect the dynamic properties of the system. This feedback between large scale structure formation and dynamics is set by the microscopic details of the competition between myosin-II filaments and crosslinking molecules. The properties of the system are sensitively controlled by the binding affinity of the myosin-II filaments to actin-fascin structures that directly regulates the maximal force the myosin-II filaments can exert. If the exerted forces are sufficiently high to disrupt actin/fascin structures, the system is dominated by the motor activity. In this regime, a highly dynamic network is formed. The dynamics in this state show pulsatile collective modes which can be attributed to the connectivity of the polydisperse structures. The pulsatile collective modes are not only found for directly connected structures but they are propagated throughout the network. They do not lead to a macroscopic contraction but persist in a dynamic steady state.

The balance of motor to crosslinker strength alone not only sets the degree of dynamics in the network but also their coordination and range. An optimal balance of motor activity and binding strength is necessary to maintain the connectivity in the network which ensures not only the occurence of pulsatile collective modes but also a highly superdiffusive dynamics. Lowering the binding affinity of the motor heads limits the internal force exertion to the crosslinked actin filaments. Consequently, the crosslinking molecules dominate relative to the motor strength resulting in a non-collective dynamics with lower superdiffusivity and a crossover to normal diffusion in a highly heterogeneous network. This opens up the possibility of inducing large scale reorganizations within an active gel, building local contractile elements or establishing the systems' heterogeneity by solely changing the maximal force the motor proteins can exert.

Any large scale reorganization within active gels depends on the intricate interplay between structure formation, aggregation and dynamic transport, which are regulated and determined by the here studied competition between force generating and force bearing structures. For large scale reorganization of the intracellular cytoskeleton active transport of the cytoskeletal building blocks, their aggregation to higher order structure are essential for ensuing force generating capabilities. The dynamics of these processes will depend on the here studied interaction and competition between the force generating and force bearing structures. The combination of simplified *in vitro* model systems with simulations based on simple interaction rules opens up the perspective of addressing the governing principles of the cytoskeletal structure formation processes.

## Materials and Methods

### Protein purification

Myosin [24] and G-actin [25], [26] are extracted from rabbit skeletal muscle (rabbit meat was obtained from *Hasenhof Weh*, Moorenweis, Germany). To fluorescently label actin, actin is dialyzed against 50 mM boric acid (pH 8.0), 0.2 mM CaCl_2_, 0.2 mM ATP and polymerized by addition of 2 mM MgCl_2_ and 100 mM KCl. F-actin is incubated with Alexa Fluor 555 succinimidylester (Invitrogen) at a molar ratio of 1∶1 at room temperature for 1 h and centrifuged at 100 000 g at 4°C for 2 h. Labelled F-actin is depolymerized by dialysis against G-buffer (2 mM Tris/HCl (pH 8.0), 0.2 mM CaCl_2_, 0.2 mM ATP, 0.2 mM DTT) at 4°C. This yields a 25% degree of labelling. Recombinant human fascin is purified from *E.coli* BL21-CodonPlus-RP and stored at −80°C in 2 mM Tris/HCl (pH 7.4), 150 mM KCl at 64 M [27].

### Fluorescence imaging

Actin is polymerized at 1 M by adding one-tenth of the sample volume of a 10-fold concentrated polymerization buffer (100 mM imidazole, 2 mM CaCl_2_, 30 mM MgCl_2_), 0.1 M myosin-II, and 1 M fascin, 20 mM creatine phosphate and 0.1 mg/mL creatine phospho kinase (Sigma) for ATP regeneration, 3 mg/mL casein to prevent any surface interactions, ATP and KCl at indicated concentrations. In this polymerization buffer, myosin-II readily polymerizes into filaments with a mean length of 0.7 m independent of the KCl concentration as determined by transmission electron microscopy. Samples are enclosed to hermetically sealed chambers to eliminate any drift in the network [7].

All data are acquired on a Zeiss Axiovert 200 inverted microscope with either a 10× (NA 0.2) long distance objective or a 40× (NA 1.3) oil immersion objective. Images are captured at 0.84 frames/s with a charge-coupled device camera (Orca ER, Hamamatsu) attached to the microscope via a 0.4× camera mount.

### Image processing

For tracing individual actin structures, images are background subtracted in ImageJ. To identify individual actin structures of the network, an intensity threshold value is applied in ImageJ to generate a binary image. Individual structures are identified as more than 10 connected bright pixels. The structures are traced over time using the IDL tracking algorithm by John C. Crocker [29] for the intensity weighted centroid positions using Matlab R2008b (The MathWorks, Inc.). To minimize tracking artefacts, the trajectories are subjected to a gliding average over 4 frames. Individual structures are traced for up to 6 min in the highly dynamic state or over 2.5 h in the passive state until they fuse with a different structures are disintegrated or move out of focus. The trajectories are divided into “runs” and “stalls”: Runs are identified as movements between two frames with velocities larger than 0.36 m/s and a change in direction smaller than 45° and the lengths of continuous runs over 2 successive frames are calculated. Run velocities are calculated as mean velocity of a single run and mean values are obtained by fitting Lorenz distributions. Mean square displacements are calculated for entire trajectories individually and the first 10% of the resulting mean square displacements are used. The power exponent 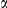 is fitted logarithmically to mean square displacements longer than 5 time points. Average values of 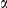 are obtained by fitting a Gaussian to the distribution of the power exponent to account for the heterogeneity of the networks. Please note, that these average values are similar but not equal to average values obtained by ensemble averaging the m.s.d.s prior to fitting as applied in [7].

To determine phases of correlated movement, the velocity cross-correlation function of moving structures 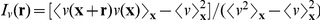 is evaluated. The degree of correlation is proportional to the average of the squared correlation function 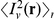 (fig. S1). The proportion of time points showing collective modes is evaluated by introducing a correlation cutoff 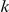: Averaged squared correlation functions 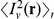 above 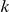 are defined as collective modes; values below 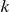 are defined to relate to an uncorrelated movement. For the data displayed in fig. 1 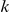 was set to 0.15. The variation of 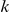 only affects the absoulte values while the relative values and the trend are not affected.

*Simulations* The experimental system consists of interconnected polar fascin bundles that are actively transported and set under stress by myosin-II filaments [7]. The microscopic processes that lead to the observed self organization, arise through the interplay of crosslinking events and active transport. More specifically, the competition between molecular motors and crosslinking proteins gives rise to the active movement, (re-)binding and forced unbinding. These microscopic interactions are the basis for numerical simulations. In a minimal approach fascin bundles are modelled as monodisperse and polar rigid rods in a quasi-two dimensional geometry. If two filaments overlap active or passive binding events occur based on probabilistic interaction rules governed by the motor and crosslinker on rates 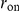 and 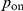. In a similar manner unbinding processes are calculated with the motor and crosslinker off-rates 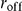 and 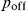. If two actin fascin bundles are actively crosslinked, torques and forces are exerted by the bipolar motor filament moving towards the plus ends of the bundles. The velocities that arise from these active crosslinks can be calculated based on a microscopic force balance of two interacting rods. This yields 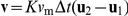 for the relative translational velocity and 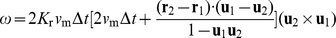 for the relative angular velocity of two actively coupled rods. The constants 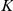 and 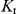 are given by the quotient of motor stiffness and the viscosities for translational and rotational movement of a rod in solution respectively, 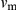 is the motor velocity and 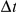 is the timestep. The vectors 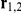 are center of mass coordinates of the polar rods and 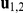 denote their orientations. The details of the derivation can be found in the supplemental online material to Ref. [7]. These expressions obey the generic symmetry-relations for motor-mediated two-filament interactions derived in Ref. [28]. Both active and passive binding events are subjected to forced unbinding. In a simplified picture this is modelled by increasing the off-rate with the number of motor proteins 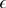 per individual cluster according to 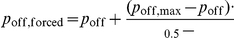, where 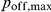 is the maximum off-rate and 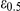 determining the rate of change of 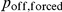. All lengths are measured in units of the rod length 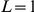. With a rod length corresponding to 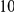 m and a motor velocity of 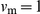 m/s, the unit of the simulation time is 1 s. If not indicated otherwise, all simulations were performed with a density 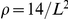, a motor on-rate 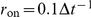, a motor off-rate 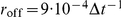, a crosslinker on-rate 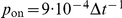 and a crosslinker off-rate 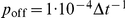. The timestep was adjusted to 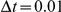 and the constants were set to 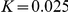 and to 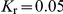 respectively.

## Supporting Information

Figure S1
**Identification and quantification of correlated modes.** First, the velocity field for the identified structures is calculated for each time point (**A**). Second the velocity cross-correlation function is evaluated for each frame. Averages of the cross-correlation function over 3 successive frames are shown in **B**. Correlation functions close to 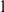 indicate highly correlated or collective movements whereas non-correlated movements average to correlation functions close to zero. If structures move in the opposite direction, they are anti-correlated with negative correlation functions. Therefore the squared correlation function, averaged over all distances 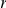, 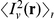 is a measure for the level of correlation at each point in time. **C** shows the time course of this averaged squared correlation function in which time points with highly correlated movements appear as peaks. To quantify the level of correlation, we introduce a global cutoff 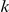. Modes in 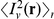 that exceed 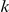 are defined as collective or correlated modes and modes with an 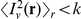 are not-correlated.(TIF)

Figure S2 **Dependence of the stall time distribution on the ATP concentration.** Low ATP concentrations (10 M, blue circles) show longest stall times, while intermediate ATP concentrations (green, 50 M, crosses or 100 M, triangles) exhibit short stalling times. At high ATP concentrations (red, 0.5 mM, open diamonds, 1 mM, squares or 2 mM, pentagrams, respectively), the stall times increase again due to the lower forces the myosin-II filaments can exert.
(TIF)

Movie S1
**Correlated dynamics of actin/fascin/myosin networks.** Active actin networks exhibit dynamic reorganizations (arrows indicate the movement of individual points in the network averaged over three frames. Lengths of the arrows are 20-fold magnified). The long range reorganizations show collective phases (velocity arrows are shown in green) during which the majority of the structures move simultaneously. The collectivity in the network is measured by the average squared velocity cross correlation function 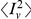. Here, phases of collective movement are reflected as peaks above 0.15 (solid black line).(MOV)

Movie S2 **Passive actin/fascin/myosin networks.** In absence of ATP, an actin/fascin/myosin network forms small and largely monodisperse clusters which move in a diffusive or subdiffusive manner.
(MOV)

Movie S3 **Dynamics at high ATP concentrations.** Active actin/fascin/myosin networks in presence of high ATP concentrations (1 mM) show dynamic reorganizations resulting in the formation of large clusters. Collective phases are not observed.
(MOV)
