# Supplementary figures and images for: Collective Dynamics of Active Cytoskeletal Networks
Source: PLoS One. 2011 Aug 26;6(8):e23798. doi: 10.1371/journal.pone.0023798 (PMC3162599; doi:10.1371/journal.pone.0023798)

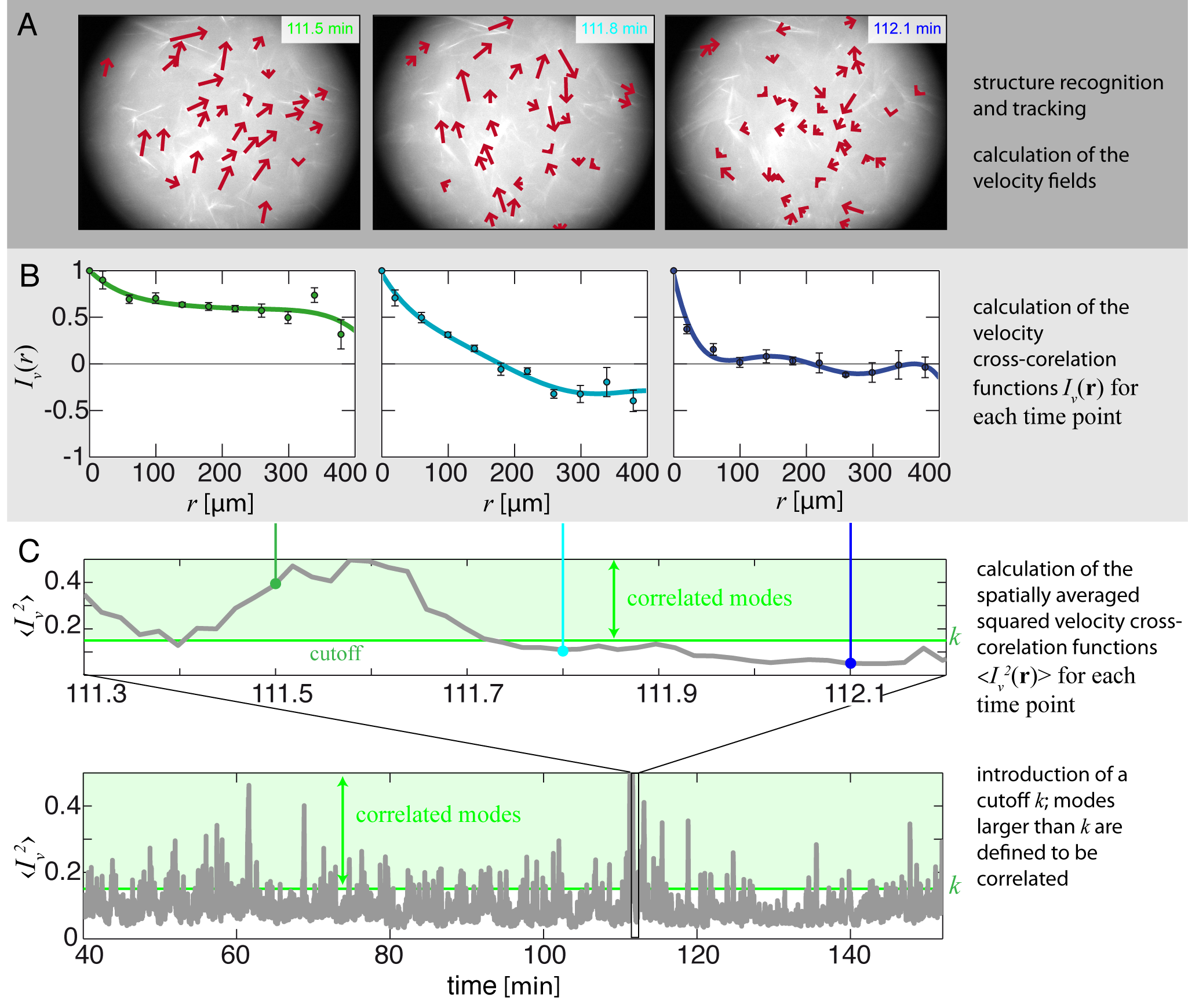

Supplement: Figure S1 — Identification and quantification of correlated modes. First, the velocity field for the identified structures is calculated for each time point (A). Second the velocity cross-correlation function is evaluated for each frame. Averages of the cross-correlation function over 3 successive frames are shown in B. Correlation functions close to indicate highly correlated or collective movements whereas non-correlated movements average to correlation functions close to zero. If structures move in the opposite direction, they are anti-correlated with negative correlation functions. Therefore the squared correlation function, averaged over all distances , is a measure for the level of correlation at each point in time. C shows the time course of this averaged squared correlation function in which time points with highly correlated movements appear as peaks. To quantify the level of correlation, we introduce a global cutoff . Modes in that exceed are defined as collective or correlated modes and modes with an are not-correlated. (TIF) [file pone.0023798.s001.tif]

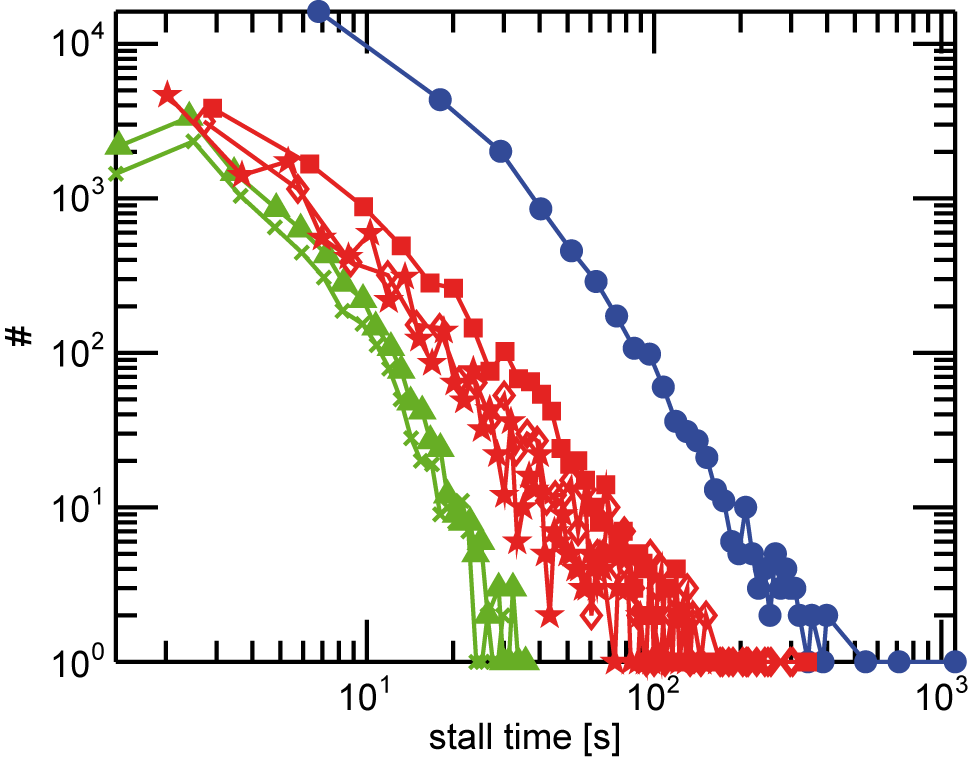

Supplement: Figure S2 — Dependence of the stall time distribution on the ATP concentration. Low ATP concentrations (10 M, blue circles) show longest stall times, while intermediate ATP concentrations (green, 50 M, crosses or 100 M, triangles) exhibit short stalling times. At high ATP concentrations (red, 0.5 mM, open diamonds, 1 mM, squares or 2 mM, pentagrams, respectively), the stall times increase again due to the lower forces the myosin-II filaments can exert. (TIF) [file pone.0023798.s002.tif]
